# Exposure of the static magnetic fields on the microbial growth rate and the sludge properties in the complete-mix activated sludge process (a Lab-scale study)

**DOI:** 10.1186/s12934-023-02207-x

**Published:** 2023-09-27

**Authors:** Ghorban Asgari, Abdolmotaleb Seid-Mohammadi, Reza Shokoohi, Mohammad Reza Samarghandi, Glen T. Daigger, Behrooz Malekolkalami, Ramin Khoshniyat

**Affiliations:** 1 Social Determinants of Health Research Center (SDHRC), Faculty of Public Health, Department of Environmental Health Engineering, Hamadan University of Medical Sciences, Hamadan, Iran; 2 Department of Environmental Health Engineering, School of Public Health, Research Centre for Health Sciences, Hamadan University of Medical Sciences, Hamadan, Iran; 3 Department of Civil and Environmental Engineering, University of Michigan, 177 EWRE Building, 1351 Beal Street, Ann Arbor, MI 48109 USA; 4 Department of Physics, University of Kurdistan, P.O. Box 66177-15175, Sanandaj, Iran

**Keywords:** Activated sludge, Static magnetic fields, Aeration reactors, α-Factor, Mixed liquor volatile suspended solids, Bioflocs

## Abstract

**Background:**

In this study, the effect of static magnetic fields (SMFs) on improving the performance of activated sludge process to enhance the higher rate of microbial growth biomass and improve sludge settling characteristics in real operation conditions of wastewater treatment plants has been investigated. The effect of SMFs (15 mT), hydraulic retention time, SRT, aeration time on mixed liquor suspended solids (MLSS) concentrations, mixed liquor volatile suspended solids (MLVSS) concentrations, α-factor, and pH in the complete-mix activated sludge (CMAS) process during 30 days of the operation, were evaluated.

**Results:**

There were not any differences between the concentration of MLSS in the case (2148.8 ± 235.6 mg/L) and control (2260.1 ± 296.0 mg/L) samples, however, the mean concentration of MLVSS in the case (1463.4 ± 419.2 mg/L) was more than the control samples (1244.1 ± 295.5 mg/L). Changes of the concentration of MLVSS over time, follow the first and second-order reaction with and without exposure of SMFs respectively. Moreover, the slope of the line and, the mean of α-factor in the case samples were 6.255 and, − 0.001 higher than the control samples, respectively. Changes in pH in both groups of the reactors were not observed. The size of the sluge flocs (1.28 µm) and, the spectra of amid I' (1440 cm^−1^) and II' (1650 cm^−1^) areas related to hydrogenase bond in the case samples were higher than the control samples.

**Conclusions:**

SMFs have a potential to being considered as an alternative method to stimulate the microbial growth rate in the aeration reactors and produce bioflocs with the higher density in the second clarifiers.

**Supplementary Information:**

The online version contains supplementary material available at 10.1186/s12934-023-02207-x.

## Introduction

Activated sludge (AS) process is the most well-known and worldwide technic for the wastewater treatment. This process has been commonly applied to remove pollutants by a microbial community of the active biomass [14, 50]. Microbial communities has a basic role on the operation of the wastewater treatment plants (WWTPs). The stability of present this kind of organisms is related to design the process, wastewater characteristics, the operational factors and, conditions of the environmental [11].

There are various modifications of AS processes for wastewater treatment, such as the complete-mix activated sludge (CMAS) process. In CMAS, the suspended growth of the microorganisms has the most critical component in the aeration reactors and, the operation of a system [7].

The aerobic methods which are used for any type of the wastewater treatment have disadvantages. Bulking of the sludge in the clarifier, due to the overgrowth of filamentous bacteria is well-known problem [10]. Rising costs of sludge disposal and, the risk of the presence of toxic materials in unsanitary disposal of solids in the environment, are the other disadvantages of this process [41].

Nowadays, for improving bio-chemical reactions, application of new combination methods such as nonosheets for active enzyems of cells [18], biochar to bioremediation of pollutants [52] and, removal contamination of the sewage sludge by use of biochar [23] have been considered by many researchers. The improvement in the AS process in terms of the higher sludge settling by a combination of the new technics is the target of many studies, too [21]. An important point in the operation of AS processes is the higher gravity settling of the bioflocs in the secondary clarifiers. Higher separation of the flocs from the liquid phase in the clarifier basins has a basic effect on the effectivness of the sludge removal [8].

The application of magnetic fields (MFs) and the growth rate of the microorganisms is an interesting discussion. Recently, studies focused on the positive effect of MFs on stimulating the growth rate of microorganisms in term of higher biodegradation of the organic components in the wastewater treatment processes [56]. A classification of MFs is according to the frequency of the electric current. If the frequency of the electric current is 0 Hz (no change is observed in the intensity overtime) the generated MFs are called static magnetic fields (SMFs) and, when the electric current has a frequency more than 0 Hz, the generated MFs are called dynamic MFs [54].

MFs are an alternative method for the treatment of wastewater [56]. Some of the fundamental properties of the liquids are viscosity, density and, tension of the surface. These components are affected when exposed to MFs that are present in the surrounding [44]. Łebkowska et al. summarized the effect of SMFs on living things in 9 categories. Changes in the properties of water, pH, higher rate of coagulation and, settling of solids are certain of them [30].

Improvement of the biomass and settling of sludge has been studied by many researchers when the application of MFs in AS is considered [39, 51]. In the process of the wastewater treatment, more growth of the microorganisms is a critical point to degradation of the organic compounds. In other words, the higher growth rate of microorganisms is equal to the higher consumption of the organic materials [56].

Many researchers are interested to use non-chemical techniques for the removal pollutants according to the side effects of the chemical materials on the environment. Non-thermal plasma catalysis [32], adsorption of the colloidal suspended particles for removal pollutants [4] and exposure of SMFs to treatment of wastewater [35] are the new aspects.

Based on available articles, there are few scientific reports on the discussion in course of the biological and the chemical changes in the components of the real CMAS process with the application of SMFs. In this case–control study, the impact of SMFs on temperature, pH, α-factor (dissolved oxygen transfer rate into the biomass), the concentration of mixed liquor suspended solids (MLSS) and, mixed liquor volatile suspended solids (MLVSS) in the aeration reactors of CMAS during the 30 days operation Lab-scale in order to estimated the role of SMFs on the optimization of oxygen transfer in the aeration reactor have been considered. Moreover, stimulate the microbial biomass growth rate and, generate the sludge flocs with higher density and, increasing the rate of the efficiency of process for removing the sludge flocs in the clarifier reactor, were the other main topics of this research.

## Materials and methods

### Source of wastewater sampling

All samples of this study were taken from the effluent of the primary clarifier of CMAS process in Sanandaj (a city located in the west of Iran) wastewater treatment plants (WWTPs) during the summer of 2022 and sent to a small feeding container (40 cm × 100 cm × 100 cm = 40 L) for distribution among the two series reactors.

### Experimental setup

**Fig. 1:**
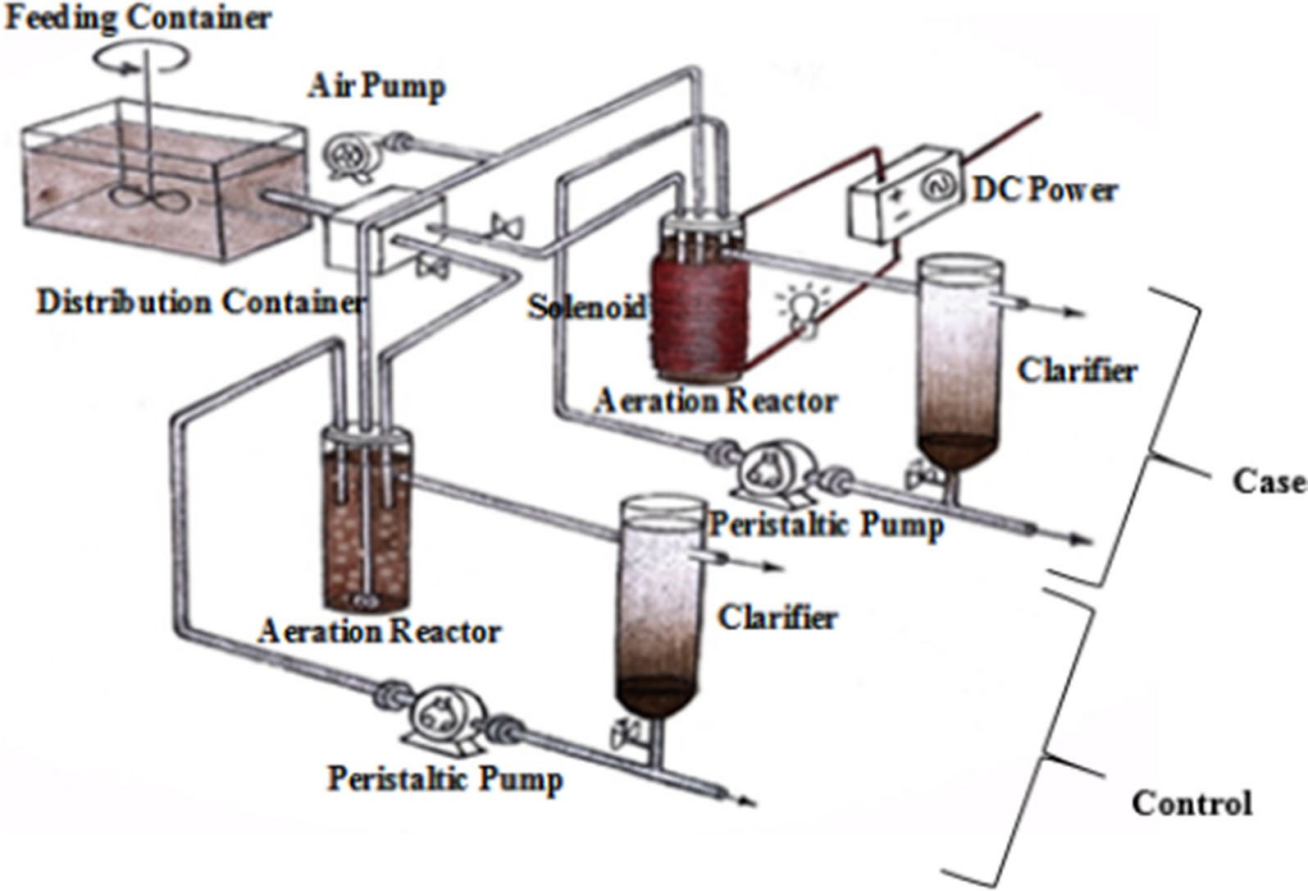
Pictorial view of devices and the reactors

The setup of this study consisted of the two series reactors (feeding and distribution containers, aeration and settling devices, air and peristaltic pumps, and sludge return systems). These devices and other accessories such as DC power, valves, and a solenoid are illustrated in Fig. 1.

It must be mentioned that, the design parameters of Lab-scale reactors (just like CMAS) were according to the real wastewater treatment plants and effluent guideline criteria [7]. Therefore, the rate of flow (mL/min) into the aeration reactors when microbial retention time was 5 days can be calculate using the following equation:

$$\mathrm{XV }=\mathrm{ YQ}{\Theta }_{c}\, A\, \, (\mathrm{S}_{0}-\mathrm{ S}) / (1+{k}_{d}{\Theta }_{c})$$

S_0_ is primary BOD_5_ (mg/L), S is the effluent BOD_5_ (mg/L), K_d_ is a kinetic coefficient which is equal to 0.06 d^−1^ and, $\Theta$_c_ is the microbial retention time (d), X is MLVSS in the aeration tank (mg/L), V is the volume of the reactors (L), Y is a degradable portion of the organic materials (kg VSS/kg COD), Q is the flow rate of wastewater (mL/min) [7].

Hydraulic retention time (HRT) was estimated using the following equation:

$$\mathrm{HRT}(\mathrm{min})={\Theta }_{c} =\mathrm{VT }/\mathrm{ Q}$$

The amount of 40 L of the wastewater from the primary sedimentation outlet of the WWTPs was transferred to the feeding reactor and distributed into the two series reactors (case and control), daily. By application of a digital pH meter (827 pH Lab Meter), a digital DO meter (HQ30D–Multi/1Channel) and, a digital infrared thermometer (Testo 831) the desired parameters were measured. The concentration of MLSS, MLVSS measured based on the standard methods, daily.

### Type of aeration system

Aeration and agitation of MLSS in the aeration reactors are carried out by an electric ambient air pump. Diffuser aeration has two main benefits in this study. Provided oxygen between 2 and 3 mg/L that was regulated by airflow meter (Yokogawa RAGL 41 Laboratory Rotameter) during the operation periods (30 days) and, mix the contents of the aeration reactors with uniform distribution of air by stone air diffusers (0.6 ± 0.1 L/min) to obtain complete-mix conditions. DO concentration in the aeration reactors was measured by DO meter (Hach HQ30D) daily.

The rate of DO concentration in the aeration basin of AS must be 1.5–4 mg/L [7].

### Static magnetic fields generation

DC power (DAZHENG PS-305D) was used for SMF_S_ generation in solenoid at the lab-scale. The number of turns of the coil was 750 rounds (0.5 mm thickness) in three rows and, they were wrapped around a sheet of the galvanized iron in order to the ever-increasing intensity of MFs. Based on the measurement by Tesla meter (GM-511 Polytronic), the intensity of generated MFs was 15 mT.

It must be mentioned that the field intensity of 15 mT was chosen because it is a intensity that can be easily produced in the laboratory using a DC power device, and it has been determined in past studies that this field intensity can stimulate the growth rate of the microorganisms.

### Seeding the aeration reactors

At the beginning of the processes, seeding of the aeration reactors as initial inoculum was done by MLSS (1750 ± 100 mg/L) from the effluent of the primery clarifier of WWTPs. For this reason, a half volume of the aeration reactors (1250 mL) was filled with MLSS. We have to do seeding the reactors because, even after 30 days of the starting the reactors without seeding, the mean concentration of MLSS in the case and control samples were only, 176.8 ± 118.9 and 120.3 ± 73.7 (mg/L), respectively.

The concentrations of MLSS that is recommended for the CMAS process are from 2500 to 6000 (mg/L) [7].

### Design of experiment

A basic parameter that has the main effect on the CMAS process is the number of the microorganisms in the bioreactor or the aeration reactor. Therefore, in terms of evaluating the efficiency of the process, the concentration of MLVSS in the aeration reactors is the key factor. The time required to reach this concentration (at least 2500 mg/L) is called start-up time and, this takes 7–28 days [7]. The flow rate of return sludge from a secondary clarifier reactor has the main effect on MLVSS concentration and performance the aeration reactors and, is calculated as the following equation:

$$100 Qr/Q = 100[(100 / pw\times SVI) -1]$$

100–150% of average flow rate is suggested to be returned to the aeration reactors in AS process [38].

In Table 1, Design parameters of CMAS reactors ($\Theta$_c_, HRT, Q, the aeration flow rate, and 100 Qr/Q) are illustrated.

**Table 1:** Design parameters of CMAS reactors

Parameter	Value	Unit
$\Theta$_c_	3	day
HRT	297	min
Q	6.7	mL/min
Aeration flow rate	0.6 ± 0.1	L/min
100 Qr/Q	%100	–

In this study, 100% of the sludge was returned to the aeration reactors by the peristaltic pumps. In Table 2. The mean of the fundamental parameters in the feeding container and, the case and control samples with and without seeding are illustrated.

**Table 2:** Mean of the main parameters in the feeding container and the case and control samples

Parametrs	Number of samples	Feeding container	Case	Control
Temperature (°C)	93	22.86 ± 0.80	29.75 ± 2.02	23.74 ± 0.70
pH	93	7.69 ± 0.25	7.80 ± 0.23	7.73 ± 0.24
DO (mg/L)	93	0.38 ± 0.15	2.6 ± 0.32	2.6 ± 0.31
MLSS (mg/L) + seeding	62	-	2260.1 ± 296.0	2148.8 ± 235.6
MLVSS (mg/L) + seeding	62	-	1463.4 ± 419.2	1244.1 ± 295.5
MLSS (mg/L) + no seeding	62	-	176.8 ± 118.9	120.3 ± 73.7
MLVSS (mg/L) + no seeddling	62	-	105.7 ± 70.8	77.6 ± 55.1

## Results and discussion

The main goal of this study was answer to this question that by exposure of 15 mT on the aeration reactor of CMAS what happened to the mean of temperature, DO concentration, oxygen mass transfer rate, pH, MLSS and MLVSS concentration in the aeration reactor and, flocs density and bonds structures of the settled sludge in the clarifiers in the case and control samples.

### Change of the temperature in the reactors

The community of the micrroorganisms in the AS is related to the optimum temperature of the environmrnt and liquied phase and has the main effect on the microbial growth rate [49]. Nevertheless, some of the researchers reported that the effect of the temperature (in the low range of changes, especially) on the category of kind and community of bacteria, is not noticeable. The community of microorganisms can be affected by other main factors, such as pH, total phosphorus concentration, and, BOD loading rate than the temperature [34, 53].

**Fig. 2:**
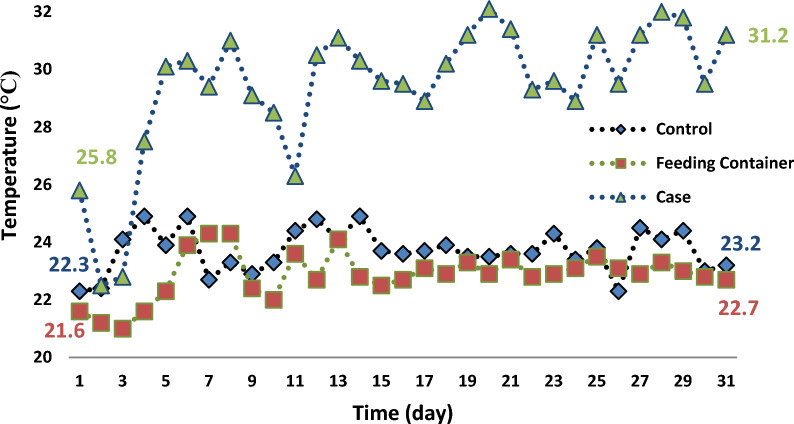
Relationship between temperature and time of the operation in the feeding container and the case and control samples

The differences between the temperature (°C) of the wastewater in the feeding container, case samples (after exposure of SMFs for one hour) and, control samples (without exposure of SMFs) during the time of the operation is demonstrated in Fig. 2.

There was a statistical difference (*p* < 0.05) between the mean temperature of MLSS in the feeding container (22.86 ± 0.80 °C) and, the case (29..42 ± 2.34 °C) samples, and, between the case and control (23.70 ± 0.74 °C) samples. However, there weren’t any differences between the mean of feeding container temperature with control samples.

In the operation of the biological wastewater treatments processes, the maintenance of temperature in the recommended ranges is the basic challenge [40]. Furthermore, as the pattern of the flow rate in our study was continuous, the mean temperature in the control samples was only 0.88 °C more than feeding container.

Due to the passage of direct current (DC) through the solenoid or wiers and, the generation of the internal resistance, heat generation is inevitable. This mechanism is known as Joule heating [31]. Since the only difference between three groups of samples was the exposure of the SMFs, it can be concluded that the application of SMFs causes the higher temperature in the case samples 6.56 °C and, 5.72 °C more than the feeding container and the control samples, respectively. However, increasing the reactor temperature can affect the dissolution of dissolved oxygen in bioreactors, too.

Finally, it seems that the interaction between the SMFs in the increasing the liquid temperature of the aeration reactors in the short period of time and, according to the continuous flow pattern rate and, keeping the concentration of dissolved oxygen between 2 and 3 mg/L during the operation of the systems in the aeration reactors, has no significant effect on the mean concentration rate of MLVSS in the case samples.

### DO concentration in the aeration reactors

The concentration of oxygen in water is called dissolved oxygen (DO) and, in the wastewater treatment processes, DO has basic role on the community and physiology of microorganisms [22]. It has been proven that DO concentration is a factor that can limit the community and distribution of the microorganisms [47]. Nevertheless, providing oxygen in the aeration reactor is one of the nain challenge and energy consumption step in WWTPs operation. Each process that could improve oxygenation efficiency, has a positive effect on the reduce the total cost of wastewater treatment [33].

**Fig. 3:**
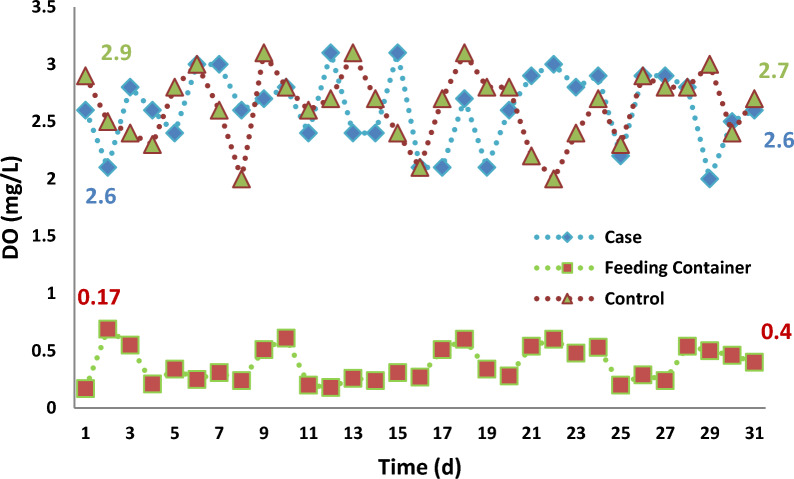
DO (mg/L) concentration in the feeding container and the aeration reactors

The mean DO concentration in the case samples was 2.6 ± 0.32 (mg/L) and, in the control, samples was 2.6 ± 0.31 (mg/L), during the operation of reactors, intentionally. The mean of this parameter in the feeding container was 0.38 ± 0.15 (mg/L). Based on statistical analysis, there were differences between the DO concentration in the feeding container compared to the case and control samples (*p* < 0.05), Nevertheless, this difference between the case and control samples was not observed (*p* = 1.000). The concentration of DO (mg/L) in the feeding container and the aeration reactors is demonstrated in Fig. 3.

One of the main aim of this study was to increase the transfer of DO from the wastewater into the biomass by applying SMFs to make the optimal use of oxygen in the aeration reactor at the proposed concentration of DO (2–4 mg/L), which is discussed in the following section.

O_2_ concentration in the aeration reactors with application of the SMFs increases, as by exposure of SMFs surface tension of the solution decresed and transmission of O_2_ from solution into the air decrease [20].

### Oxygen mass transfer (OMT)

Some parameters can affect the rate of oxygen mass transfer (OMT) into the biomass in the aeration processes, such as biomass concentration, type and rate of the aeration, hydrodynamic qualification, solids retention time (SRT) and, biofilms conditions [15, 46]. OMT and volumetric OMT coefficient (K_L_a) are the two most important parameters to evaluate biofilms quality in the wastewater treatment processes [17, 33]. In this way, K_L_a and α-factor (0.25–0.65) are used to estimate the OMT quality in the wastewater [42].

In AS process, MLSS (mg/L) is the main propellant agent for controlling K_L_a and α-factor. MLSS must be between 10 and 15 g/L to have a basic efficiency in the transfer of oxygen into the biomass [33]. The range of α-factor on air diffusers is 0.3–0.85 [6].

α-factor can be estimated from the following equation:

$${\upalpha }= \frac{{\mathrm{K}}_{\mathrm{L}}\mathrm{a }\,(\mathrm{process \,water})}{{\mathrm{K}}_{\mathrm{L}}\mathrm{a }\,(\mathrm{clean \,water})}$$

K_L_a is a volumetric oxygen transfer coefficient (1/h).

α-factor can be estimated according to SRT as following equation:

$$\mathrm{\alpha-factor }= 0.019\mathrm{ SRT}+0.533\pm 0.093$$

As consumption of substrate by the microorganisms happened during the operation of the systems, and increasing SRT, so the rate of α-factor increases with increasing SRT [5]. Following equation is suggested to measure this relationship [19]:

$$\mathrm{\alpha-factor }= 0.51-0.062\mathrm{ MLVSS}+0.019\mathrm{ SRT}\pm 0.114$$

**Fig. 4:**
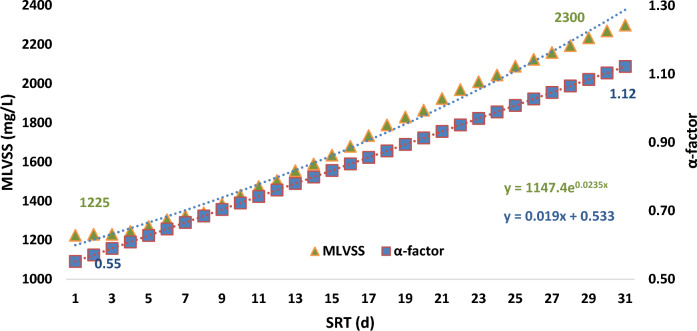
Relationship between MLVSS, α-factor and SRT in the case samples

There is a linear relationship between the α-factor, and SRT. The relationship between three main parameters (MLVSS, α-factor and SRT) in the case samples of the aeration reactor is shown in Fig. 4.

The correlation between MLVSS (g/L) and α-factor is positive non-linear equation (y = 1147.4e^0.0235x^). However, this correlation with SRT (d) is positive and linear equation (y = 0.019x + 0.533).

**Fig. 5:**
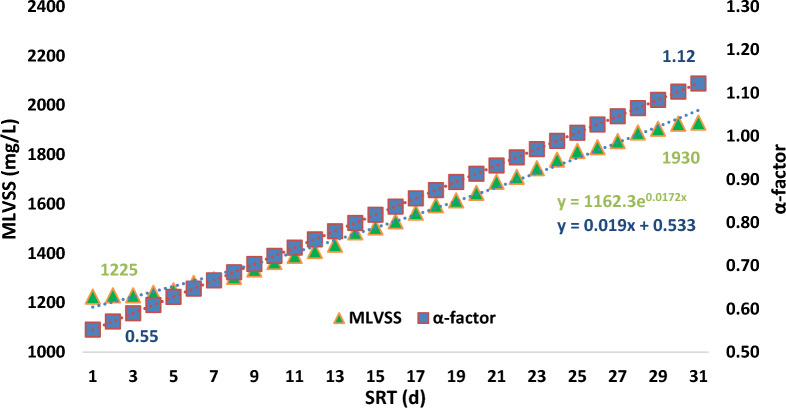
The relationship between MLVSS, α-factor and SRT in the control samples

In Fig. 5 the relationship between MLSS, α-factor, and SRT in the control samples is demonstrated.

The patern of the relationship between the MLVSS, α-factor, and operation time of the system in the control samples was look like the case samples, too. By exposure of MFs in the biological processes, the rate of oxygen transfer into the cell of the microorganisms increases. This impact was on the increasing amount of α-factor. At the beginning of the processes, the amount of α-factor in both reactors was 0.45 and over time increased. However, the increase of α-factor at the end of one-month operation of the systems in the case reactor was 0.96 and, in the control samples was 0.98 (slop of line in both groups was 0.019). The mean of α-factor in the case samples only 0.01 unit was less than the mean of that in the control samples.

The aeration method in WWTPs is a costly process (about 15–49% of total energy consumed by a plant) and, saving energy, especially in the discussion of the increasing the efficiency of oxygen to generate higher biomass, must be considered [12]. Nowadays, the operation of the aeration reactors with a low concentration of DO for saving energy suggested [13].

Baquero-Rodríguez et al. argued that the concentration of TSS (total suspended solids) has fundamental effect on the α-factor. when it was less than 6 g/L there is a positive relationship between them and, higher than 6 g/L of TSS has negative effect on the α-factor [6]. Inhibitory of substances in term of the transfer oxygen into the biosorption, suggested for this limitation [1].

### Changes of pH in the aeration reactors

Water characteristics such as electrical conductivity and pH could be affected by exposure of MFs [36].

As MFs stimulated the rate of the bacterial growth rate in the wastewater, a fall in pH in the solution or the external of bacteria (such as *E. coli*) by decomposition of glucose, happened [45]. So it seems that the changes in wastewater pH are unimpressive when the intensity of SMFs is 15 mT and the flow pattern is continuous.

**Fig. 6:**
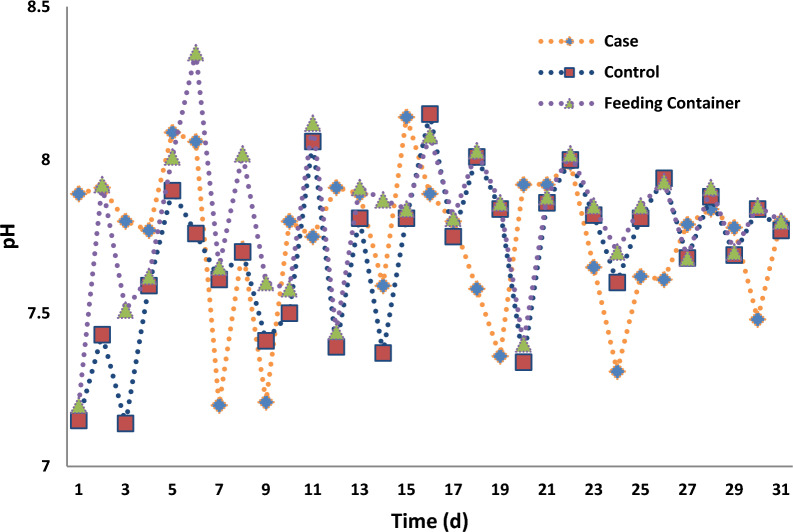
Changes in pH during 30 days of the operation in feeding container, the case and control samples

Based on statistical analysis, no significant changes were observed in the pH of all samples in the aeration reactors (p > 0.05). Changes in pH value during 30 days of the operation in the feeding container, the case, and, control samples in the aeration reactors are illustrated in Fig. 6.

It must be considered that the intensity of 15 mT of SMFs for one hour in the continuous flow pattern of CMAS, was not in such a way to cause major changes in the pH of the solution between the case and, control samples. According to this, there is no need to adjust the pH of MLSS in the aeration reactors, as the SMFs did not change the pH of aeration contents more than the recommended range.

Moreover, the typical pH for the most biological processes is 6–9 [7].

### Mixed liquor suspended solids (MLSS)

**Fig. 7:**
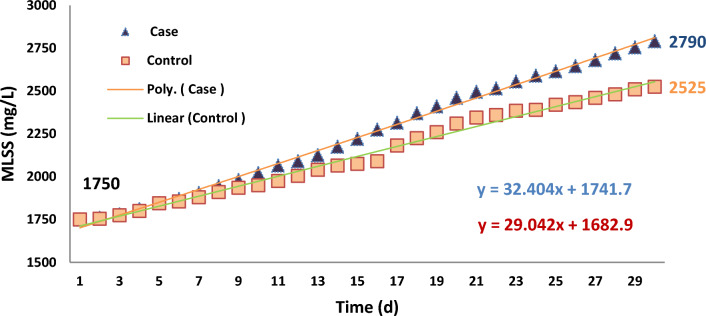
MLSS concentration in the case and control samples

According to the result of the analysis of the data in terms of evaluation of MLSS in the aeration reactors, the concentration of MLSS (mg/L) in the case and, control samples was not statistically different (p = 0.107), as shown in Fig. 7. Nevertheless, In both reactors, the concentration of MLSS increased, daily.

Improve of MLSS concentration in the aeration reactors is based upon first-order kinetic, however, changes of this concentration in the reactor with the application of SMFs were similar to second-order kinetic.

**Fig. 8:**
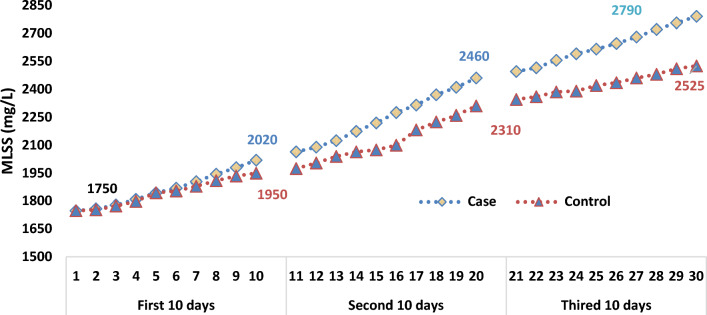
Correlation between concentration of MLSS (mg/L) and time of the operation

At the beginning of the experiments (first 10 days), the changes in MLSS concentration were similar in both reactors. Moreover, a trend changes of MLSS concentration in the second and third 10 days was growing. This correlation is illustrated in Fig. 8.

**Fig. 9:**
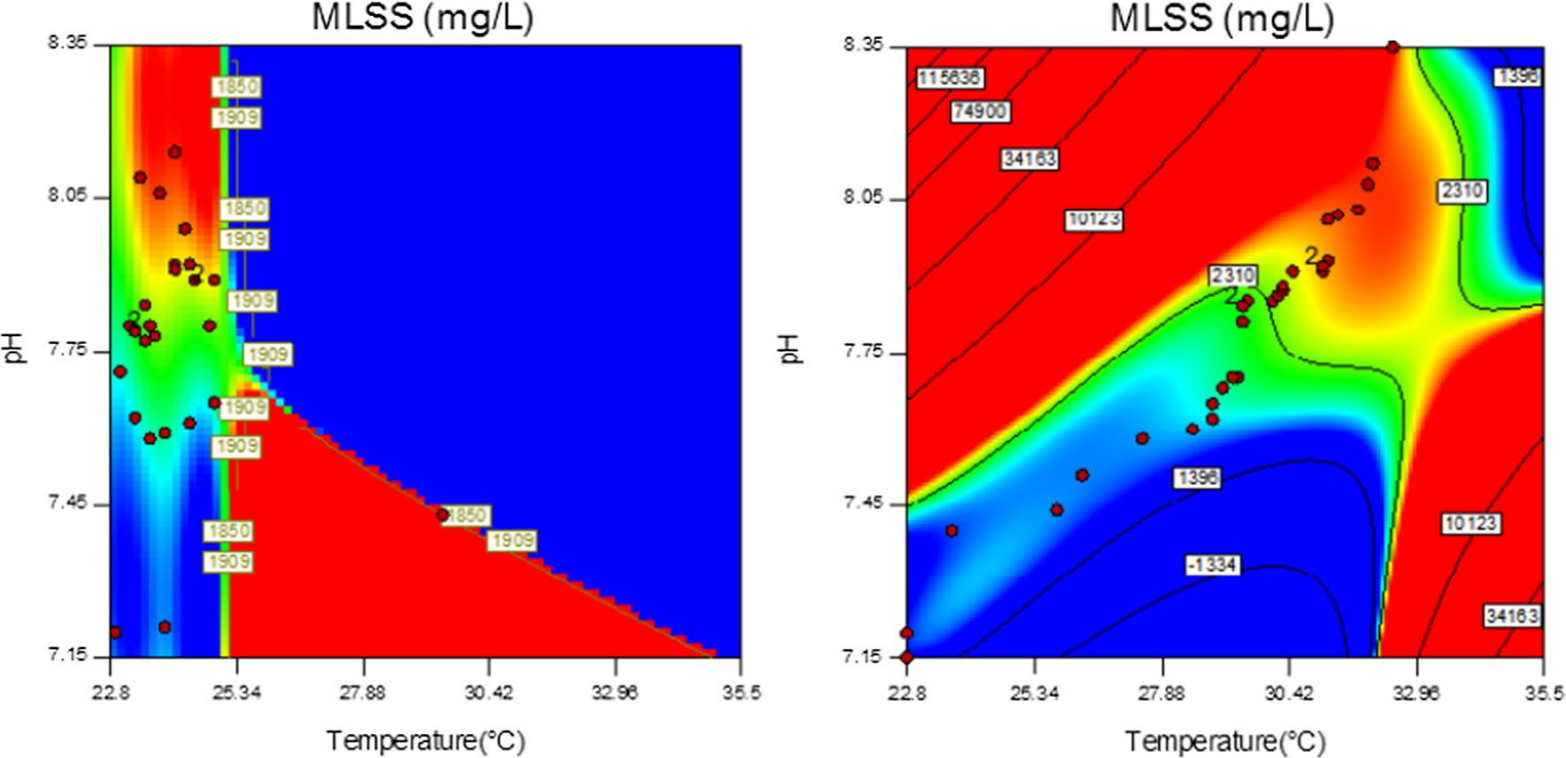
Relationship between MLSS, pH and temperature of the case (left) and the control samples (right)

The relationship between MLSS, pH, and temperature in both reactors is illustrated in Fig. 9. The red dots of the places indicate the concentration of MLSS. As it is clear, at a temperature of about 30 (°C) and pH around 7.8, the concentration of MLSS was more than the other area in the case samples (Fig. 9 on the left). Nevertheless, all the red dots (concentration of MLSS) for the control samples, accumulated at temperatures under 25.34 (°C) and pH around 7.75 (Fig. 9 on the right).

Zieliński et al. in their research reported that SMFs could improve MLSS in the aeration reactors of AS by about 470 ± 20 mg/L in the case samples (3420 ± 710 mg/L) higher than the control samples (2950 ± 670 mg/L) [56]. The mean concentration of MLSS in the case samples of SBR (sequencing batch reactor) was 1.4 (g/L) more than the control samples. In our study the mean concentration of MLSS in the case and control samples was 1689 ± 351.0 and 1535 ± 235.5 (mg/L). In other words, the differences between the mean concentration of MLSS in the case and control samples was 154 (mg/L) that the most of them was happened after 10th day of the operation of systems.

### Mixed liquor volatile suspended solids (MLVSS)

Most of the portion of MLVSS is related to bacteria and the organiv materials. In AS process, the density of the microorganisms in the aeration reactor can be estimated by measurement of the concentration of MLVSS (mg/L), approximately [25, 26].

**Fig. 10:**
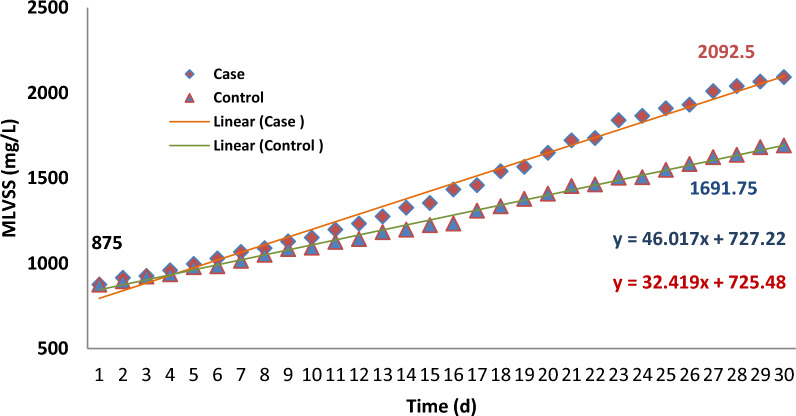
Relationship between MLVSS (mg/L) and time of operation in the case and control samples

In Fig. 10. effect of SMFs (15 mT) on MLVSS in the case samples compared to the control samples, was illustrated. Although the changes in MLVSS at the beginning of the process were imperceptible, simultaneously, the difference between the mean of MLVSS in two groups of samples increased and becomes statistically significant, over time (p < 0.05).

The correlation between the concentration of MLSS in both groups (the case and control) was not statistically significant. Moreover, in terms of MLVSS, this difference is significant (*p* = 0.45). The SMFs, have the properties to affect on the growth rate of living components of MLSS, not the non-living components. The relationship between the improvement of MLVSS over time in both groups (case and control) is shown in Fig. 10.

There was a linear correlation relationship between MLVSS and the time of the operation of the systems in the control samples. Nevertheless, the paterrn of the growth rate of MLVSS in the case samples was look like a second-order reaction. Therefore, it can be said that the rate of growth in the mean concentration of the microorganisms or MLVSS in the case samples (1.44E3 ± 397.7 mg/L) was higher than in the control samples (1.26E3 ± 257.9 mg/L).

The correlation between MFs and the growth rate of microorganisms is not linear [57].

**Fig. 11:**
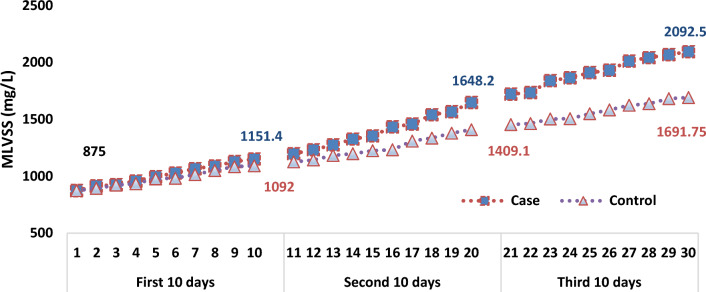
Trend of MLVSS (mg/L) concentration changes during the operation of system in the case and control samples

It must be mentioned that the changes of MLVSS concentration in first 10 days of the operation in the case and control was similar with each other as shown in Fig. 11.

**Fig. 12:**
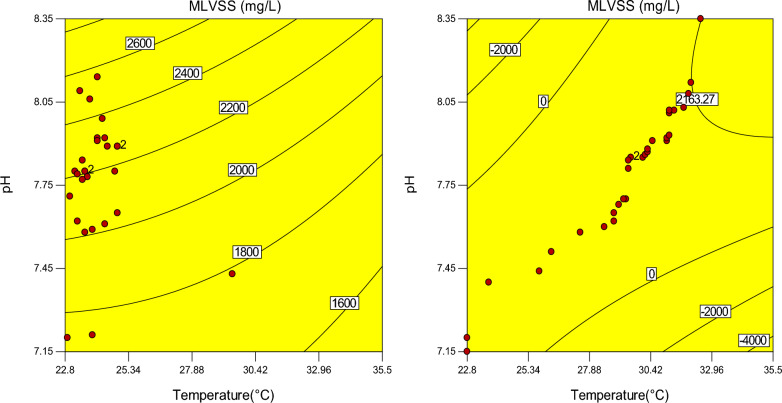
Contour line diagram (pH, temperature and MLVSS) for the case (left) and control samples (right)

In the second 10 days of the operation of the system, increasing the concentration of the MLVSS (mg/L) was observed. To determine the changes in trend and a correlation between temperature, pH value, and concentration of MLVSS, contour line diagrams have been used. In the case samples, the red dots were gathered around a pH of 7.89 and, temperature was gathered between 28.62 and 32.5 (°C) as shown in Fig. 12 on the left. These trends and correlations of pH and temperature are illustrated for the control samples, too (Fig. 12 on the right). As specified, the accumulation of data was between 22.8 and 25.32 (°C) and pH 7.75.

It seems that, despite the positive and incremental trend changes of MLVSS in the case samples, when compared to the control samples, no changes occurred in the pH of both groups. One main reason for this is related to the pattern of flow. As a continuous flow pattern was used in our study, changes in pH in two groups of the samples were not significant.

Shuhao Huo et al. reported that the use of MFs could improve the highest generation of biomass of microalga (4.44 g/L) at 30 °C [24]. In our study the more growth of MLVSS in the soludtion in the case samples was around 30 °C.

Enzymes stimulation and, improve of the microbial community by exposure of 11.4 mT SMFs could increase 85.5% of microbial counts in the case samples [55].

### Flocs density and bonds structures

**Fig. 13:**
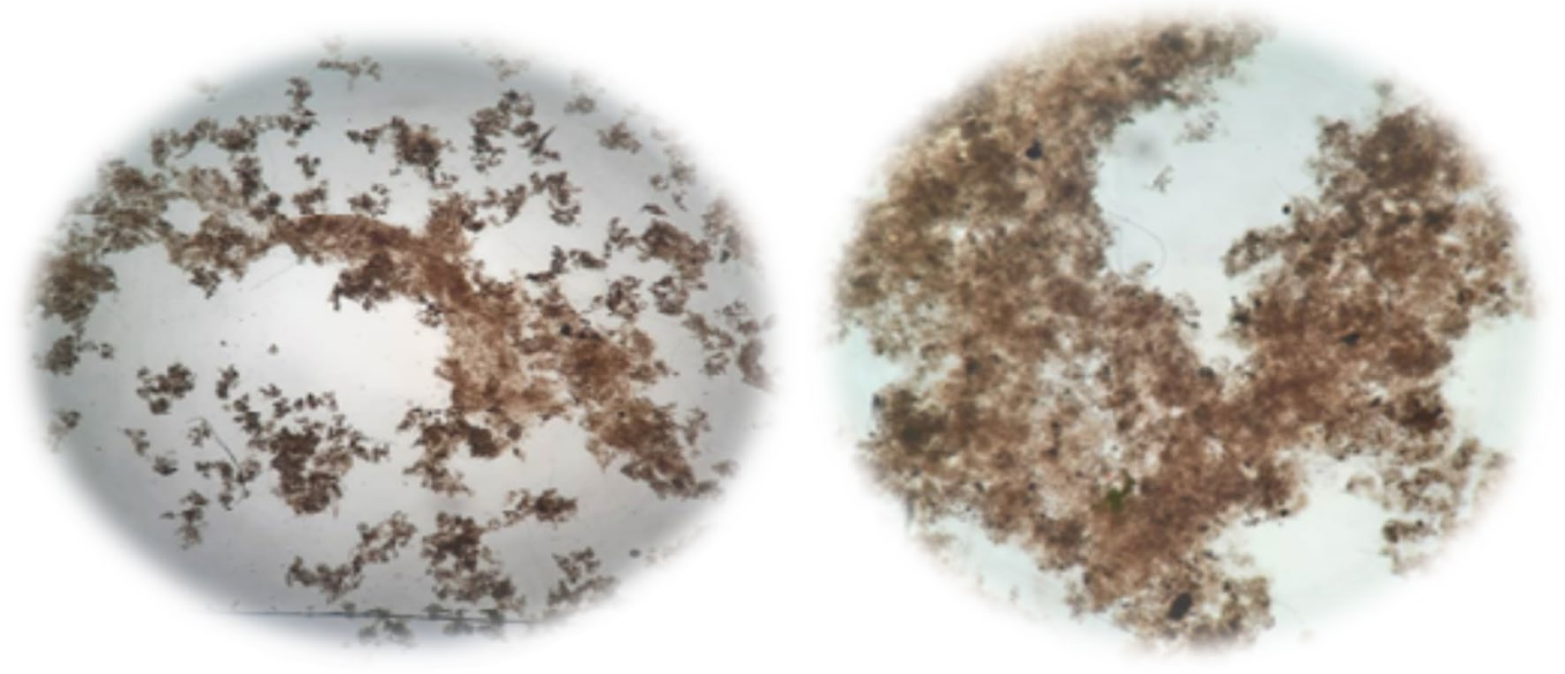
Light microscopic image (1000 × magnification) of the flocs in the case (left) and control (right) samples

In Fig. 13 the flocs structure (1000 × magnification) at the outlets of the aeration reactors in the case and the control samples on the 30th day of the operation of systems by light microscope, is illustrated.

Microscopic image is one method to determine the generation of the microbial flocs in AS processes [27]. Quality and segments of the flocs can be observed by this method in the wastewater samples [28]. As it is clear, the volume and density of the flocs on the case sample was higher than the control sample.

**Fig. 14:**
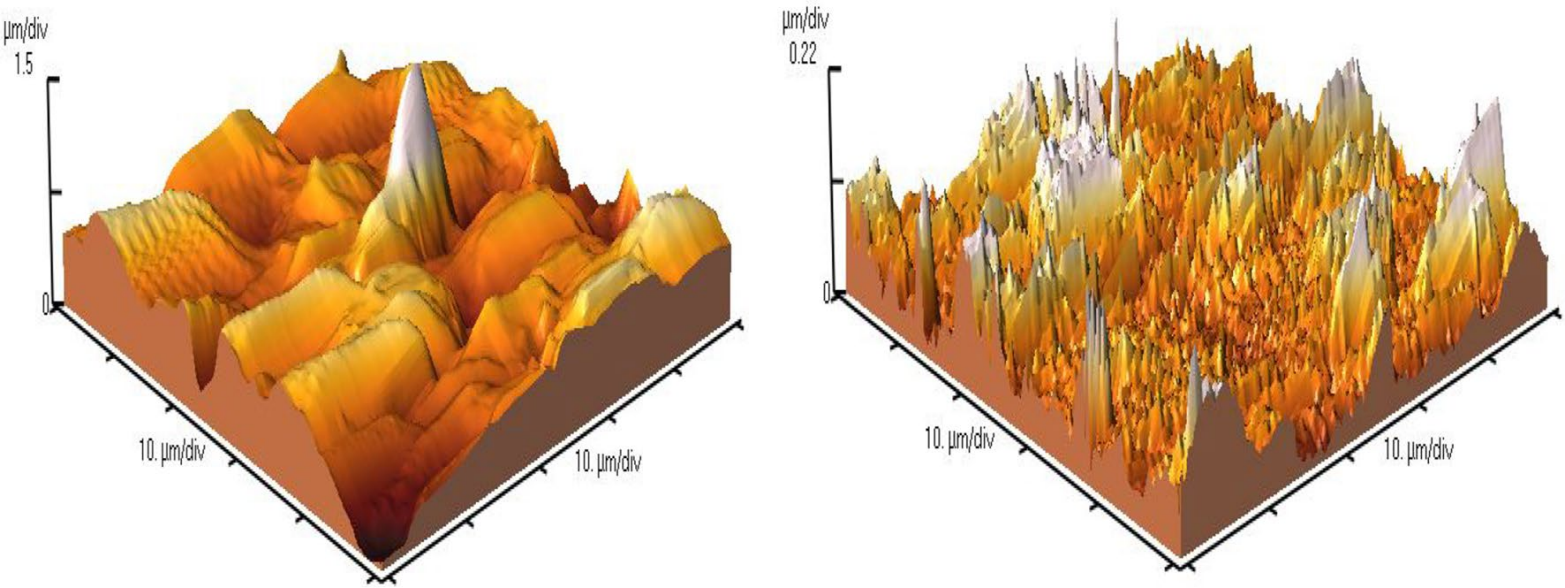
Size of the flocs in the case (left) and control (right) sample by AFM

In Fig. 14 image of *Atomic Force Microscopy* (AFM) in the case and, control samples on the 15th day of the operation of the system is illustrated.

AFM scan is a new three dimentional image to observed surface roughness and smoothness of the particles and, size of them [29].

SMFs could improve the density of the flocs in the aeration reactors of CMAS when the intensity of SMFs was 15 mT [2]. MFs can improve sedimentation of the sludge due to an inhibitory effect on the growth rate of filamentous bacteria [57].

Generated the low volume of the sludge by sludge dewatering methods in WWTPs is a key factor that has foundamental effects on the cose of the operation [52].

In addition to the generation of the higher density of flocs by SMFs, the size of flocs increased, too. By application of 15 mT SMFs for 1 h daily on the aeration reactor, density of flocs could be increased. An interesting point in this section of the study was that the surface of the flocs when exposed to SMFs was rough. Nevertheless, the surface of the flocs without the application of SMFs was not so uneven (Fig. 14).

**Fig. 15:**
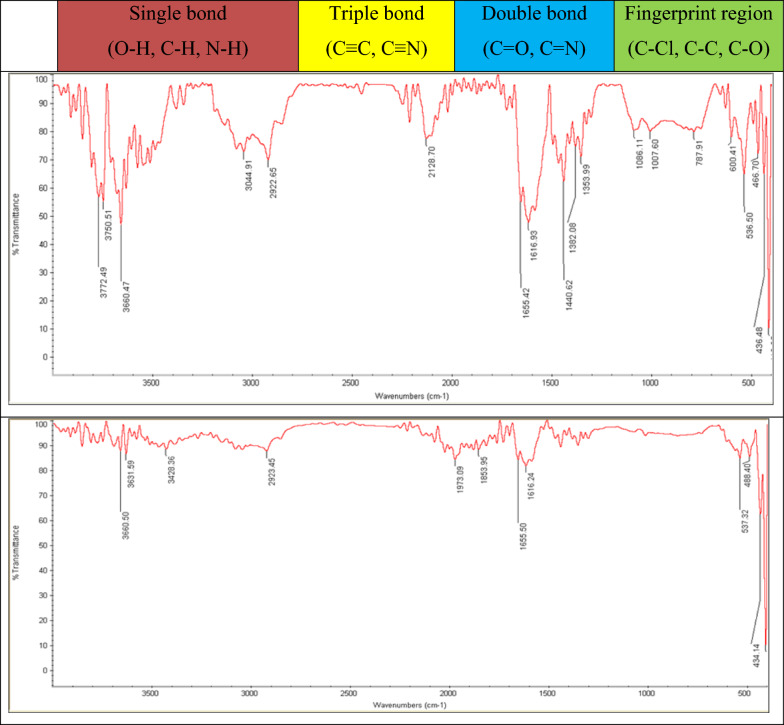
FTIR spectra of the flocs in the output of settled sludge in the clarifier reactors in the case (up) and the control samples (down)

In order to determine chemical changes in the sludge properties, FTIR (*Fourier-transform infrared spectroscopy*) spectra was used for the flocs in output of the settled sludge in the secondary clarifier reactors in two groups of samples that were demonstrated in Fig. 15.

FTIR analysis is a method to determine the functional variation of the chemical groups in the flocs of MLSS. Based on analyses of wavenumbers, the percent of transmittance in the sharp peaks of the case (17 sharp peaks) was higher than the control (10 sharp peaks) sample in all bonds of the chemical substances. These sharp peaks indicated that the density of materials in the case samples was higher. It can be concluded that by application of SMFs (15 mT) the ultimate target of the wastewater treatment in the aeration reactors is obtained by generating a higher density of the flocs for higher settling in the clarifier.

Ren et al. reported that intensity of 15–25 mT of MFs improves the metabolism of bacteria by the effect of more generation of dehydrogenase and, more consumption of substrate [43]. In this study, The spectra of amid I' (1440 cm^−1^) and II' (1650 cm^−1^) areas related to hydrogenase bonds in the case samples were higher than the control samples and, this indicates that the microbial growth rate in the case samples in which the SMFs applied was higher due to higher production of hydrogenase enzyme.

Deamici et al. in 2019 reported that the use of MFs method have many adventages for instant, no secondary pollution, low cost and, nontoxic material. They also, mentioned to stimulate of cell metabolism and more carbohydrates synthesis [9].

### Energy consumption

One of the basic dissadventages of AS processes is more use of the electrical energy for the apply oxygen into the aeration reactors [16]. In WWTPs 40–75% of the total of the electrical energy consumer is used to the aeration of wastewater [37]. Moreover, application of the MFs for treatment of wastewater have adventages such as improve an efficient, easy-to-use method and, low-cost [48].

In our study we use 150 W of alternative current (220 V) of the electrical energy daily, for supply SMFs (Additional file 1).

For reduce energy consumption in this course of the wastewater treatment scale use of the solar panel during the day, is recommended [3].

## Conclusion and outlook

The higher growth rate of the microorganisms in the aeration reactors to the transformation of the soluble organic matter into the flocs and removal of BOD_5_ is a basic point of the operation of the CMAS process. The supply of oxygen required for the metabolism of the organic matter in the aeration reactors has always been one of the most challenges in the operating of the aeration system due to the need for higher energy consumption. Furthermore, as oxygen solubility in water is low, the use of new methods (such as the application of SMFs) that can increase the efficiency of the aeration process (an alternative method for the process of the wastewater treatment in the aerobic conditions). Application of 15 mT intensity of SMFs on the aeration reactor could effect of the α-factor as the basic parameter in term of the transition of oxygen into the MLVSS and, generation the higher concenteratuon of MLVSS in the case samples. Furthermore, Higher settling of the flocs in the secondary clarifier under the exposure of SMFs is related to the higher density of them, is the main key to improving the effectiveness of the seconed clarifier. By the application of 15 mT intensity of SMFs in the wastewater treatment processes, a new view of the use of the physical methods can be achieved without consuming the chemical materials.

Effect of SMFs on components of the rectors were limited to development of MLVSS. Although MLVSS is one vital part of MLSS, the impact of SMFs on MLSS was not significant. The reason for this issue may be due to the presence of the microbial mass in MLVSS as a portion of MLSS.

The effect of SMFs on α-factor or rate of oxygen transfer is stimulated. Even when the concentration of DO was adjusted by a uniform flow rate during the processes. In other words, the SMFs cause a more efficient use of oxygen in the aeration reactors.

By the applicaton of SMFs in the aeration reactor of the case sample, the size of the generated flocs decreased based on the AFM image and, the sharp peaks of amids related to dehydrogenase bond as the indicator of the microbial growth rate, was higher than the control sample.

### Supplementary Information

**Additional file 1**. Aeration and clarifiers reactor (Case sample); Aeration and clarifiers reactor (Control sample); Case and control samples reactors.

## Data Availability

On request from the corresponding author.

## Abbreviations

AC: Alternative current
AS: Activated sludge
AFM: Atomic Force Microscopy
BOD: Biological oxygen demand
CMAS: Complete-mix activated sludge
COD: Chemical oxygen demand
DC: Direct current
DO: Dissolved oxygen
FTIR: Fourier-transform infrared spectroscopy
HRT: Hydraulic retention time
k_d_: Endogenous decay coefficient
K_L_a: The volumetric oxygen transfer coefficient
MFs: Magnetic fields
MLSS: Mixed liquor suspended solids
MLVSS: Mixed liquor volatile suspended solids
OMT: Oxygen mass transfer
Q_r_/Q: The recycle ratio
S_0_: Primary substrate concentration
S: Effluent substrate concentration
SMFs: Static magnetic fields
SRT: Solids retention time
SVI: Sludge volume index
WWTPs: Wastewater treatment plants
X: Biomass concentration
Y: Maximum biomass yield coefficient
Θ_c_: Microbial retention time
ρ_w_: Density of water
