# Supplementary material for: Exposure of the static magnetic fields on the microbial growth rate and the sludge properties in the complete-mix activated sludge process (a Lab-scale study)
Source: Microb Cell Fact. 2023 Sep 27;22:195. doi: 10.1186/s12934-023-02207-x (PMC10523802; doi:10.1186/s12934-023-02207-x)
Supplement: Supplementary file 1 — Additional file 1. Aeration and clarifiers reactor (Case sample); Aeration and clarifiers reactor (Control sample); Case and control samples reactors. [file 12934_2023_2207_MOESM1_ESM.docx]

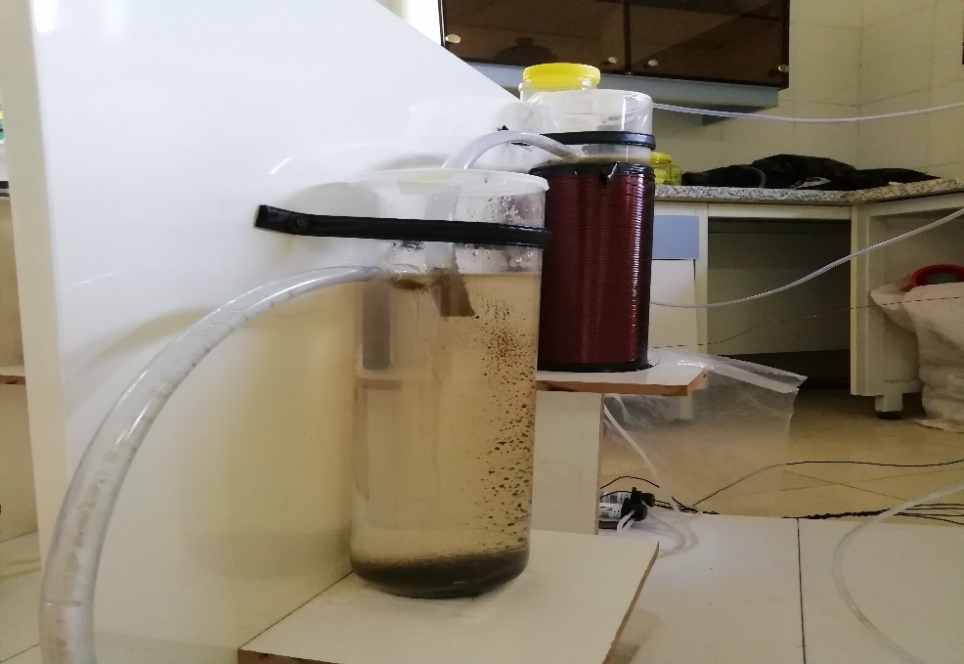


Aeration and clarifiers reactor (Case sample)


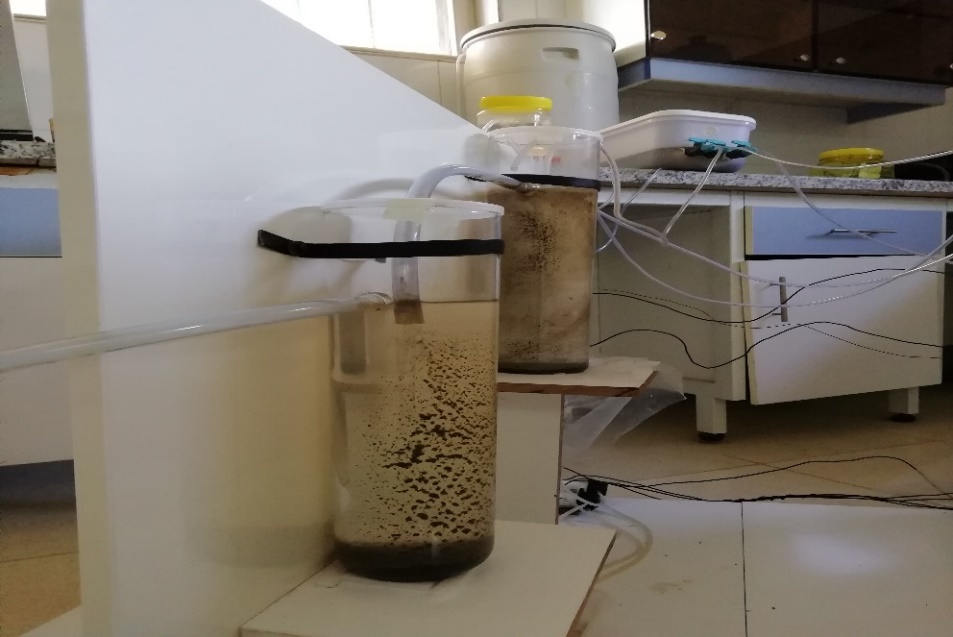


Aeration and clarifiers reactor (Control sample)


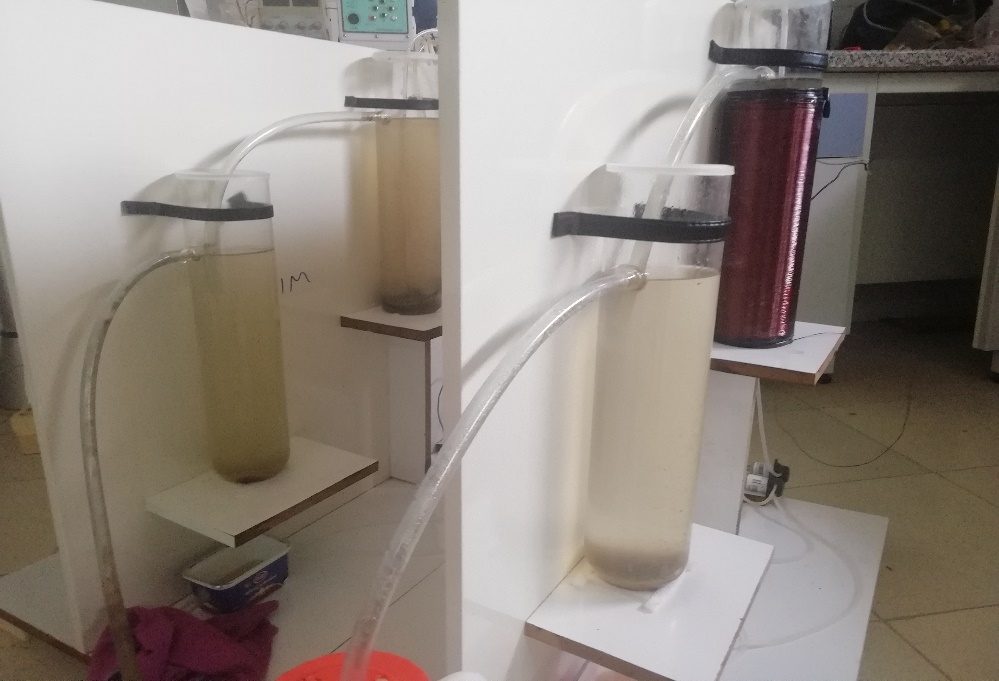


Case and control samples reactors
